# Development of monosodium acetate-induced osteoarthritis and inflammatory pain in ageing mice

**DOI:** 10.1007/s11357-015-9792-y

**Published:** 2015-05-14

**Authors:** Andrea C. Ogbonna, Anna K. Clark, Marzia Malcangio

**Affiliations:** Wolfson Centre for Age-Related Diseases, King’s College London, Guy’s Campus, London Bridge, London, SE1 1UL UK

**Keywords:** Osteoarthritis, Pain, Microglia

## Abstract

Most conditions associated with ageing result from an age-related loss in the function of cells and tissues that maintain body homeostasis. In osteoarthritis (OA) patients, an inadequate response to stress or joint injury can lead to tissue destruction which can result in chronic pain. Here, we evaluated the development of monoiodoacetate (MIA)-induced OA in 3-, 15- and 22-month-old mice and assessed the pain-like behaviours and the spinal microglial changes associated with MIA administration. We observed that in aged mice, nocifensive behaviour was significantly attenuated in comparison to young adults despite similar knee joint pathology. Specifically referred mechanical allodynia associated with the MIA initial inflammatory phase (0–10 days) was significantly attenuated in 22-month-old mice. In contrast, the late phase of MIA-induced mechanical allodynia was comparable between age groups. Significant increase of microglia cell numbers was detected in 3, but not 15- and 22-month-old spinal cords. Furthermore, in the zymosan model of acute inflammation, mechanical allodynia was attenuated, and microglial response was less robust in 22 compared to 3-month-old mice. This study suggests that nocifensive responses to damaging stimuli are altered with advancing age and microglial response to peripheral damage is less robust.

## Introduction

The main risk factor for osteoarthritis (OA) is age, and people over 65 years commonly display radiographic changes of OA, particularly osteophytes, in their joints (Goekoop et al. 2011). Clinical OA is defined by features in patient history and upon examination, requiring the presence of joint pain (Oliveria et al. 1995; Jinks et al. 2007). Chronic pain conditions are more prevalent in the elderly, and pain perception is altered with ageing (Gibson and Farrell 2004). As there are currently no disease-modifying therapies for OA, treatment is aimed at managing the symptoms of pain and swelling, trying to minimise functional impairment to maintain quality of life. Analgesics that are currently recommended for treatment of OA pain include paracetamol, NSAIDs, opioids and cyclooxygenase (COX)-2-selective inhibitors (coxibs) (Eccles et al. 1998; Geba et al. 2002; Jawad 2005).

Most conditions associated with ageing result from an age-related loss in function of cells and tissues to maintain body homeostasis, especially when placed under stress. In OA patients, an inadequate response to stress or joint injury can lead to joint tissue destruction. How this joint destruction might then result in pain development can also be affected by age; both peripheral and central components of the nociceptive pathway show age-related changes (Gibson and Farrell 2004). Indeed, peripheral nerves in aged subjects display altered functional, structural and biochemical properties such as slower axonal regeneration after injury (Verdu et al. 2000). There are also age-related changes centrally that result in hyperexcitability within the dorsal horn of the spinal cord and impaired descending modulation (Iwata et al. 2002). Both neuronal sensitisation at the site of joint damage in the periphery (peripheral sensitization) (Schaible et al. 2009) and centrally in the dorsal horn of the spinal cord (central sensitization) contribute to chronic pain mechanisms (Schaible 2006). OA is considered as a progressive degenerative process which is tightly integrated with inflammation. Therefore, the age-related alterations to the peripheral and central sensory pathway offer reasoning behind the increased incidence of painful chronic conditions such as OA. Peripheral inflammation is associated with sensitisation of nociceptive afferent fibres in the joint by pro-inflammatory mediators which results in reduced threshold of fibre activation, increased firing and increased fibre input centrally in the dorsal horn of the spinal cord. The responsiveness of spinal dorsal horn neurons to mechanical and heat stimuli applied to the periphery, background activity, after-discharge and receptive fields are significantly enhanced after inflammation (Schaible and Schmidt 1988; Grubb et al. 1993; Iwata et al. 1999; Iwata et al. 2002). Besides central neuron sensitization, a microglial response in the spinal cord has been observed in models of peripheral inflammation (Svensson et al. 2003; Clark et al. 2007; Staniland et al. 2010; Clark et al. 2012). Similarly, spinal and brain microglia acquire an activated morphology during normal ageing (Streit and Xue 2009) and can release pro-inflammatory mediators capable of enhancing nociceptive transmission.

In this study, we employed a mouse model of OA in which a single injection of monoiodoacetate (MIA) is delivered to the knee joint, inducing peripheral changes that include local acute inflammation followed by cartilage erosion, joint disruption and increased pro-inflammatory cytokine expression in the joint (Bove et al. 2003; Guzman et al. 2003; Fernihough et al. 2004; Orita et al. 2011). There is also evidence of central changes that include spinal cord neuronal and microglial activation, as well as increased nociceptive neuropeptide release evoked by activation of the central terminal of primary afferents into the dorsal horn (Ogbonna et al. 2013; Rahman and Dickenson 2015). The contribution of microglial activation is suggested by the observation that repeated administration of the microglia inhibitor, minocycline, attenuated pain behaviour and reduced the number of activated microglia in the spinal cord of MIA-treated rats (Sagar et al. 2011).

Specifically, in the current study, we evaluated the development of MIA-induced OA in 3-, 15- and 22-month-old mice with the following two objectives: first, to assess the pain-like behaviours associated with MIA administration. Second, to assess potential changes of spinal microglial response which is considered a measure of central activity after peripheral damage.

## Materials and methods

### Animals

All experiments were carried out in accordance with UK Home Office Regulations (Animal Scientific Procedures Act, 1986) using male adult C57Bl/6 mice aged 3, 15 or 22 months old (20–40 g, Charles River, UK). Food and water were available ad libitum, and mice were housed under standard conditions with a 12-h light/dark cycle for a week prior to behavioural experiments to acclimatize. Experimental study groups were randomized, and experiments were performed by an observer unaware of treatments.

### Monosodium iodoacetate model of osteoarthritis

As previously described (Ogbonna et al. 2013), mice were anaesthetised by isoflurane/O_2_ inhalation and received a single intra-articular injection into the left knee of 1 mg of monosodium iodoacetate (MIA) (Sigma, UK) in a total volume of 10 μl of physiological sterile saline. The left leg was flexed at a 90° angle at the knee, and the solution was injected through the intrapatellar ligament using a 30-G needle and a Hamilton syringe. Control mice received an intra-articular injection of sterile saline (10 μl). In our previous study, we established that 1 mg MIA induces pain-like behaviour from 3 days from intra-articular injection (Ogbonna et al. 2013). At 4 weeks from MIA injection, the knee joints display loss of cartilage and proteoglycan staining compared to contralateral and control knee joints (Ogbonna et al. 2013).

### Intraplantar zymosan model of inflammatory pain

As described previously (Staniland et al. 2010), the intra-plantar zymosan model of inflammation consisted in the injection of 20 μl of zymosan (0.2 mg/ml in saline; Zymosan A from *Saccharomyces cerevisiae*; Fluka Analytical, Germany) into the intraplantar surface of the right hind paw. The time interval of 24 h after injection was selected to investigate pain behaviour. Zymosan was injected under isofluorane (Abbott Animal Health, UK) inhalation anaesthesia using a 500-μl U-100 microfine insulin syringe with a 29 gauge needle (Becton, Dickinson & Co, UK). Mechanical (von Frey hairs) and thermal (Hargreaves method) nociceptive thresholds were measured on three occasions (baseline) prior to and 24 h after zymosan injection.

### Behavioural testing

#### Weight bearing asymmetry

Changes in weight bearing were assessed as a measure of primary pain-related hypersensitivity. The weight borne by each hind limb was measured using an Incapacitance Tester (Linton Instruments, Norfolk, UK) as previously described (Ogbonna et al. 2013). Mice were placed in a Perspex chamber designed so that their two hindpaws were resting on separate transducer pads. Once the mice were settled in the correct position, a reading of their weight distribution over the two paws was taken over 3 s. Results were calculated as weight borne on the ipsilateral hindlimb as a percentage of total weight borne by the mouse using the formula: [ipsilateral weight borne / (ipsilateral + contralateral weight borne)] × 100. A value of 50 % represents an equal weight distribution across ipsilateral and contralateral hindlimbs, and a value of less than 50 % is indicative of a reduction in weight borne on the ipsilateral hind limb. Two baseline trials were recorded prior to injection of MIA/vehicle. Mice were subsequently assessed on days 3, 7, 10, 14, 17, 21 and 28 after injection. Each session consisted of three separate trials separated by at least 5 min.

#### Mechanical hypersensitivity

Static mechanical withdrawal thresholds were assessed by applying calibrated von Frey hairs (0.008–1 g, Touch Test, Stoelting, Wood Dale, IL, USA) to the plantar surface of the hindpaw according to the ‘up-down’ method (Chaplan et al. 1994), as previously described (Ogbonna et al. 2013). Unrestrained mice were acclimatized in acrylic cubicles (8 × 5 × 10 cm) on a wire mesh grid, giving access to the underside of their paws, for up to 60 min prior to testing. Calibrated von Frey hairs were then applied to the plantar surface of the hindpaw until the fibre was bent. The hair was held in place for 3 s or until the paw was withdrawn in a reflex not coupled to movement or grooming. Each test started with application of the 0.6-g filament (middle of the series of 10 hairs), and each hindpaw was assessed alternately. If the mouse withdrew its paw, the next lower force was applied and vice versa until there was a change in response from the mouse. Four successive hairs were then assessed according to the up-down sequence with no hair filament applied more than three times in order not to sensitize the paw. The 50 % paw withdrawal value was calculated using the method described by Dixon (1980). Three baseline measurements were made prior to administration of MIA/vehicle, and mice were then subsequently assessed on days 3, 7, 10, 14, 17, 21 and 28 after injection.

#### Thermal hypersensitivity

Heat-pain threshold of the hind paw was determined with the Hargreaves method using the Plantar Test (7370; intensity 50; Ugo Basile, Italy). Unrestrained animals were acclimatised in acrylic cubicles (8 × 5 × 10 cm) atop a uniform glass surface for up to 60 min prior to testing. An infrared light source was directed onto the plantar surface of the hind paw, and the latency to paw withdrawal was automatically measured in seconds. Three responses were recorded for each hind paw on each testing occasion with at least 1 min between stimuli. To avoid tissue injury, the maximum stimulus latency was 23 s.

#### Behavioural analysis

All data were analysed using SigmaPlot 11 (Systat Software Inc, UK) and statistically compared using two-way repeated measure (RM) ANOVA followed by Student Newman Keuls post hoc test. Area under the curve data were analysed by two-way ANOVA followed by Student Newman Keuls post hoc test. Data are shown as mean ± SEM, and *p* < 0.05 was set as the level of statistical significance.

#### Knee processing and histology

At the end of the behavioural studies, 4-week post-injection of 1 mg MIA or vehicle (saline), mice were deeply anaesthetized with sodium pentobarbital then transcardially perfused with heparinized (1 U/ml) saline followed by 4 % paraformaldehyde in 0.1 M phosphate buffer. The knees were dissected out, and surrounding muscle was trimmed. Tissues were post-fixed overnight and then placed in decalcifying solution (AlCl_3_ · 6H_2_O 7 % *w*/*v*; formic acid 5 % *v*/*v* and HCl 8.5 % *v*/*v* in distilled water) for a maximum of 24 h. The decalcified knee joints were washed overnight in 0.1 M phosphate buffer pH 7.4 and then processed to paraffin wax. For toluidine staining, 10-μm-thick sections of tissue were cut and mounted onto slides. Sections were then de-waxed in xylene, rehydrated in descending concentrations of alcohol, followed by washing in distilled water before staining in 0.05 % toluidine blue (aq) for 5 min. Sections were then rapidly dehydrated in four changes of absolute alcohol, cleared in xylene and mounted under coverslips using DPX mounting medium (VWR, UK). Histological and anatomical evidence of joint pathology was not quantified.

#### Immunohistochemistry

At the end of the behavioural studies, 4-week post-injection of 1 mg MIA or vehicle (saline), mice were deeply anaesthetized with sodium pentobarbital then transcardially perfused with heparinized (1 U/ml) saline followed by 4 % paraformaldehyde in 0.1 M phosphate buffer. Lumbar spinal cord were dissected out and post-fixed for 2 h before being transferred to 20 % sucrose solution (VWR) in 0.1 M phosphate buffer for 48 h at 4 °C. Tissue was mounted in optimum cutting temperature embedding medium (VWR) then snap frozen with liquid nitrogen and stored at −80 °C until further processing. Transverse spinal cord sections (20 μm) were cryostat cut and thaw mounted onto Superfrost plus microscope slides (VWR). Slide-mounted spinal cord sections were incubated overnight with primary antibody solution for rabbit anti-ionized calcium-binding adaptor molecule 1 (Iba-1; 1:1000, Wako Chemicals, Neuss, Germany), followed by fluorescent secondary antibody solution for 2 h (IgG-conjugated Alexa Fluor 488, Invitrogen Molecular Probes, Carlsbad, CA, USA). All antibody solutions were prepared in PBS with 0.1 % Triton X-100 (BDH, VWR, Lutterworth, UK) and 0.2 % sodium azide (Sigma, UK). All slides were coverslipped with Vectashield Mounting Medium containing nuclear marker 4 ′,6-diamidino-2-phenylindole · 2HCl (DAPI; Vector Laboratories, Peterborough, UK), and fluorescent staining was visualized using a Zeiss Axioplan 2 fluorescent microscope.

#### Quantification and analysis of immunohistochemistry

In order to determine whether microglial cell number in the dorsal horn was altered in response to peripheral damage, we performed quantitative assessment of Iba-1 immunoreactivity in spinal cord sections from MIA, zymosan and saline treated mice was carried out by counting positive profiles within the dorsal horn (average area 3 × 10^5^ μm^2^ encompassing laminae I–V or average area 1 × 10^4^ μm^2^ encompassing laminae I–III). Three spinal cord sections were evaluated per animal, with a minimum of four animals per group, and the experimenter was blind to treatment throughout the duration of the quantification process. All data were analysed using SigmaPlot 11 (Systat Software Inc, UK) and statistically compared using two-way ANOVA followed by Student Newman Keuls post hoc test. Data are shown as mean ± SEM, and *p* < 0.05 was set as the level of statistical significance.

## Results

### Age-related effects on MIA-induced weight bearing asymmetry and mechanical allodynia

**Fig. 1:**
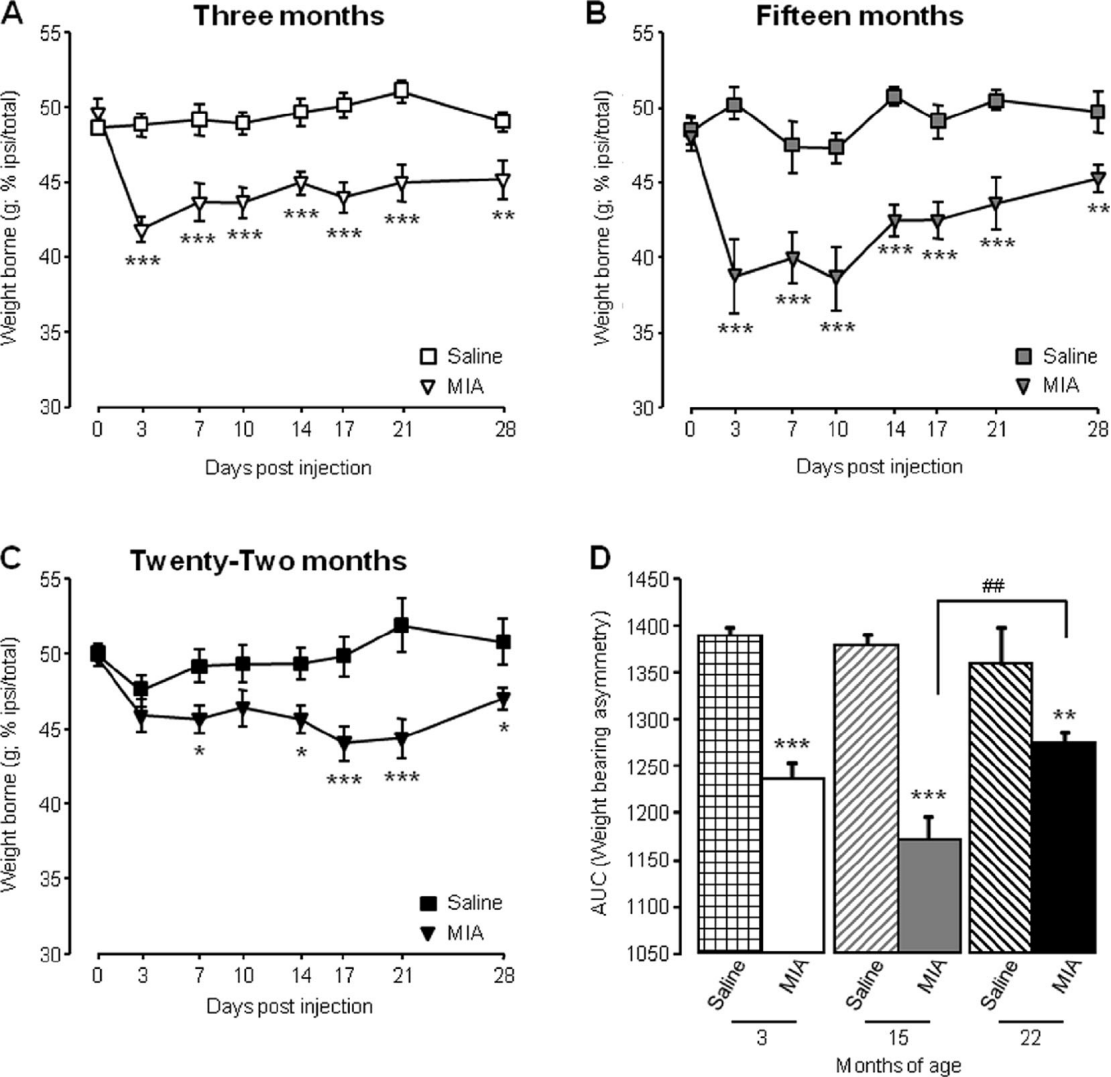
Ageing affects MIA-associated weight bearing asymmetry. **a**–**c** Weight borne on ipsilateral hind limb was monitored at regular intervals in 3-month (**a**), 15-month (**b**) and 22-month (**c**)-old mice before and until 28 days following intra-articular injection. ***p < 0.001, **p < 0.01, *p < 0.05 compared to saline control group, two-way RM ANOVA post hoc Student Newman-Keuls. **d** Area under the curve (AUC) analysis of weight borne ***p < 0.001, **p < 0.01 compared to age-matched saline control; ##*p* < 0.01 between 15- and 22-month-old mice; two-way ANOVA, post hoc Student Newman-Keuls. Data shown as mean ± SEM, *n* = 10–16 mice per group

As we previously reported (Ogbonna et al. 2013), following intra-articular administration of MIA 3 month old mice developed significant weight bearing asymmetry compared to saline-treated mice, evidenced by a reduction in weight borne on the ipsilateral hind limb, peaking at day 3 (42 ± 0.8 %) and persisting for the duration of the study until 28 days after injection (Fig. 1a). Furthermore, area under the curve analysis (AUC) showed a significant reduction in AUC for 3-month-old mice injected with MIA compared to those injected with saline (Fig. 1d). When the weight bearing capacity of 15-month-old mice was tested after intra-articular injection, those injected with MIA also developed significant weight bearing asymmetry compared to saline-treated mice, with the greatest reduction in ipsilateral weight borne on day 10 (37 ± 2.5 %) (Fig. 1b). This was supported by a reduction in AUC values of 15-month-old MIA injected mice compared to age-matched saline-treated mice (Fig. 1d). In contrast, MIA-associated weight bearing changes were attenuated in 22-month-old mice (Fig. 1c). Peak reduction in ipsilateral weight borne did not occur until day 17 (44 ± 1.1 %) and AUC analysis indicated that allodynia developed significantly less that in 15-month-old mice (Fig. 1d).

**Fig. 2:**
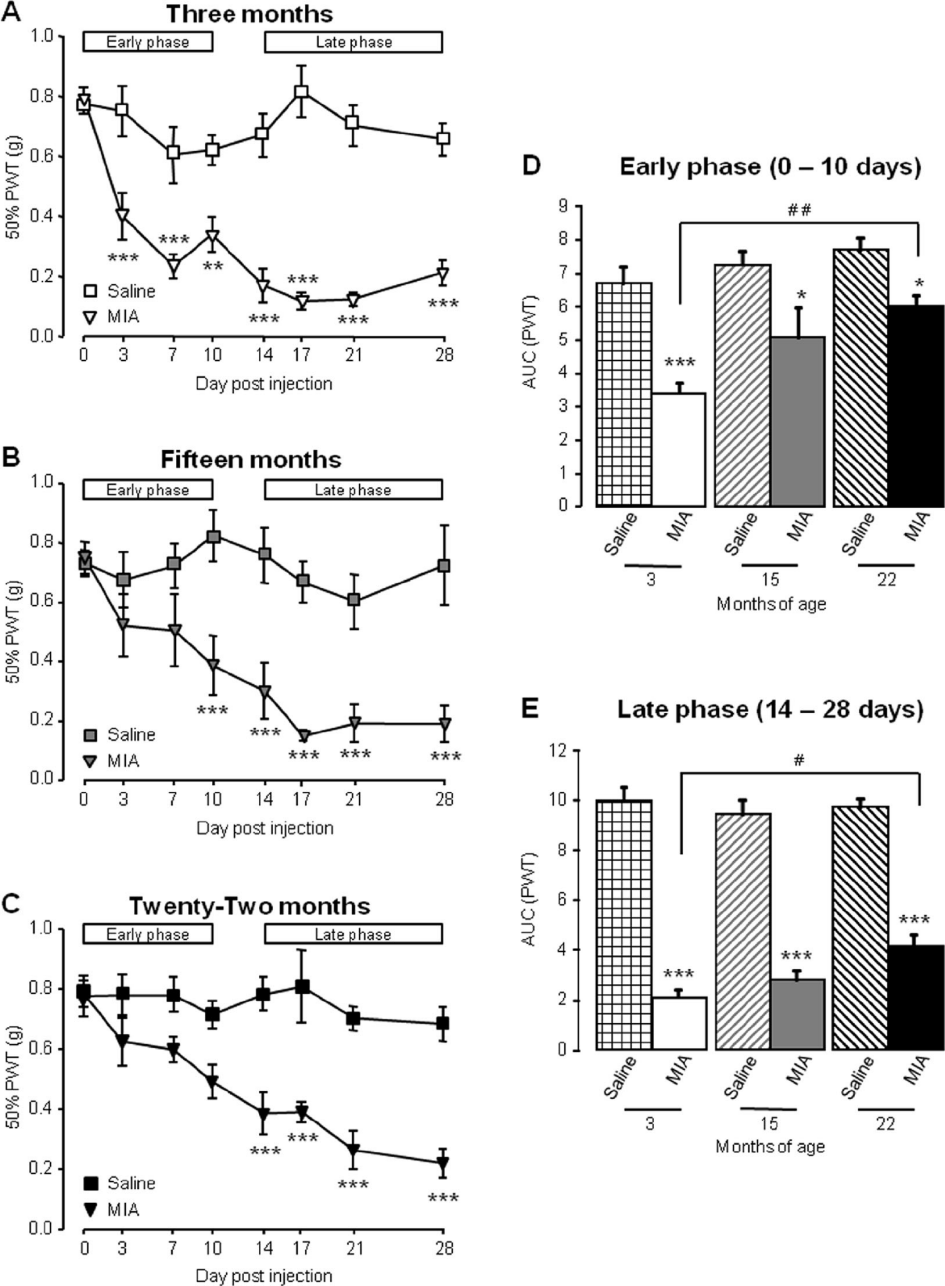
Ageing affects MIA-associated referred mechanical hypersensitivity. **a**–**c** Mechanical withdrawal responses of ipsilateral hind paws were measured before and at regular intervals until 28-day post-intra-articular injection in 3-month (**a**), 15-month (**b**) and 22-month (**c**)-old mice*. White bars* represent the duration of the early and late phases of mechanical hypersensitivity. ***p < 0.001, **p < 0.01 compared to saline control group, two-way RM ANOVA, post hoc Student Newman-Keuls (**d**–**e**); AUC was calculated for all groups from 0 to 10 days (early phase; **d**) and from 14 to 28 days (late phase; **e**). ***p < 0.001 compared to age-matched saline control group, ## *p* < 0.01; #*p* < 0.05 between groups as indicated, two-way ANOVA, post hoc Student Newman-Keuls. Data are shown as mean ± SEM, *n* = 10–16 mice per group

MIA injection into the mouse knee is associated with referred mechanical hypersensitivity in the ipsilateral hind paw that occurs in a biphasic manner with an early (0–10 days) and late (14–28 days) phase (Ogbonna et al. 2013). Here, this observation was confirmed in 3-month-old mice with an initial reduction in paw withdrawal thresholds (PWT), peaking at day 7 and a second reduction that peaked at day 17 (Fig. 2a). AUC analysis on the early and the late phase of mechanical hypersensitivity demonstrated that MIA group values were significantly lower than the saline group values in 3-month-old mice (Fig. 2d, e). Interestingly, for both 15- and 22-month-old mice, there was no significant reduction in PWT until day 10 and day 14, respectively (Fig. 2b, c). The initial mechanical hypersensitivity observed in 3-month-old mice was reduced in both groups of aged mice, evidenced by significant difference in the AUC values of MIA-treated mice compared to saline controls in 22-month-old mice during the early phase (Fig. 2d). In contrast, during the late phase, there was a significant reduction in AUC values for MIA-treated mice compared to saline across all age groups (Fig. 2e).

These data demonstrate that the extent of MIA-induced pain-like behaviour is attenuated in aged mice, compared to young mice. Specifically, 15- and 22-month-old mice develop an attenuated early phase of mechanical hypersensitivity which is thought to represent the inflammatory phase of the model (Fernihough et al. 2004).

### MIA-induced cartilage degradation in the knee joint of aged mice

**Fig. 3:**
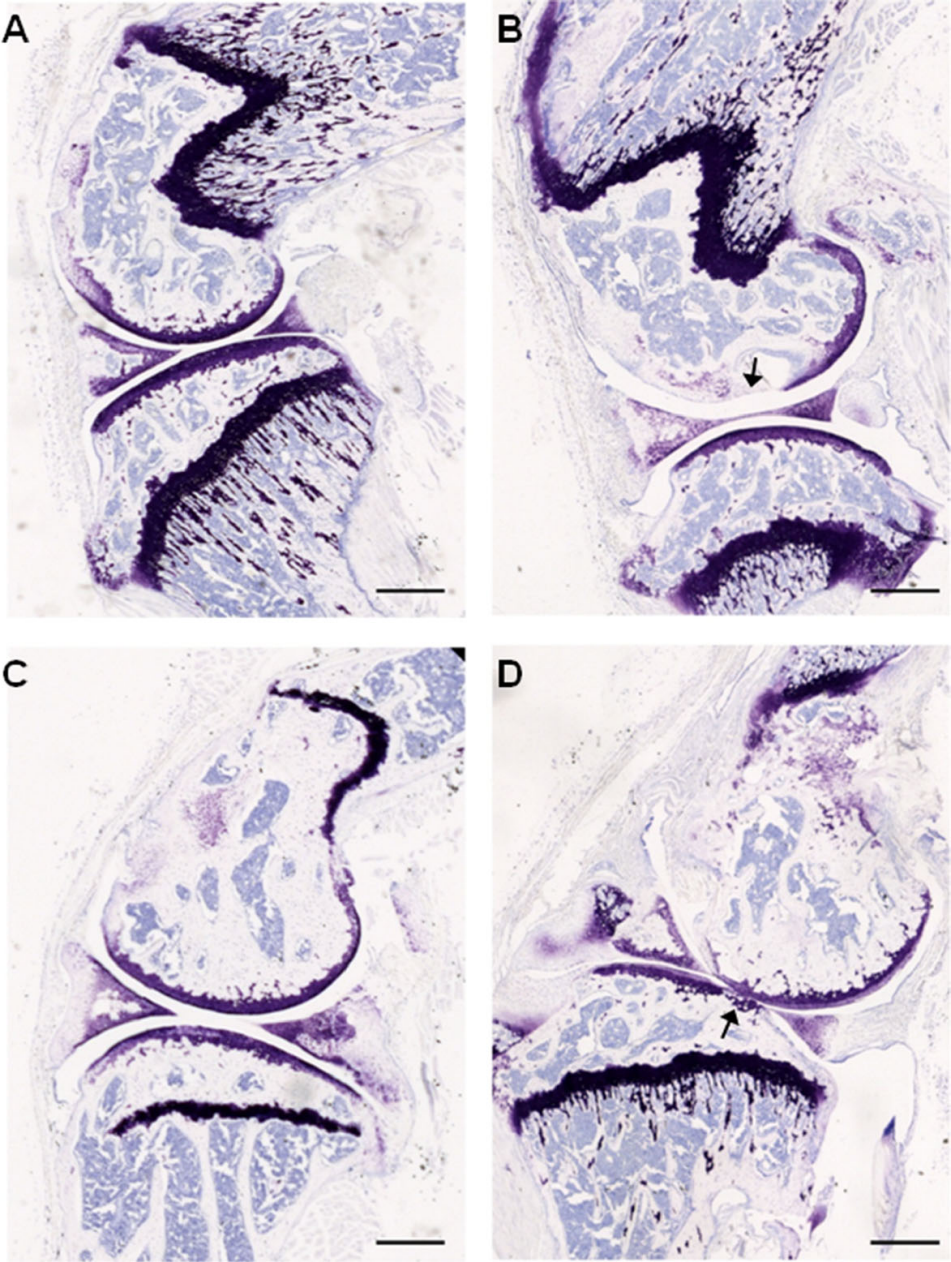
Intra-articular MIA administration results in knee joint pathology in young and aged mice. Toluidine blue staining of articular cartilage from saline (**a**, **c**) and MIA (**b**, **d**) injected ipsilateral knee joints. Smooth and intact articular surface of the femur and tibia from 3-month (**a**) and 22-month (**c**)-old mice injected with saline in contrast to thinning and loss of articular integrity (indicated by *arrows*) in 3-month (**b**) and 22-month (**d**)-old mice injected with 1-mg MIA. *Scale bar* = 500 μm

Histological examination by way of toludine blue staining of knee joints 28 days following intra-articular injections demonstrate that saline-treated knees from both 3- and 22-month-old mice have intact and smooth articular surfaces (Fig. 3a, c). MIA-treated knees from 3- to 22-month-old mice displayed thinning cartilage and loss of proteoglycan staining (Fig. 3b, d; shown with arrows).

### Age-related effects on MIA-induced microgliosis

**Fig. 4:**
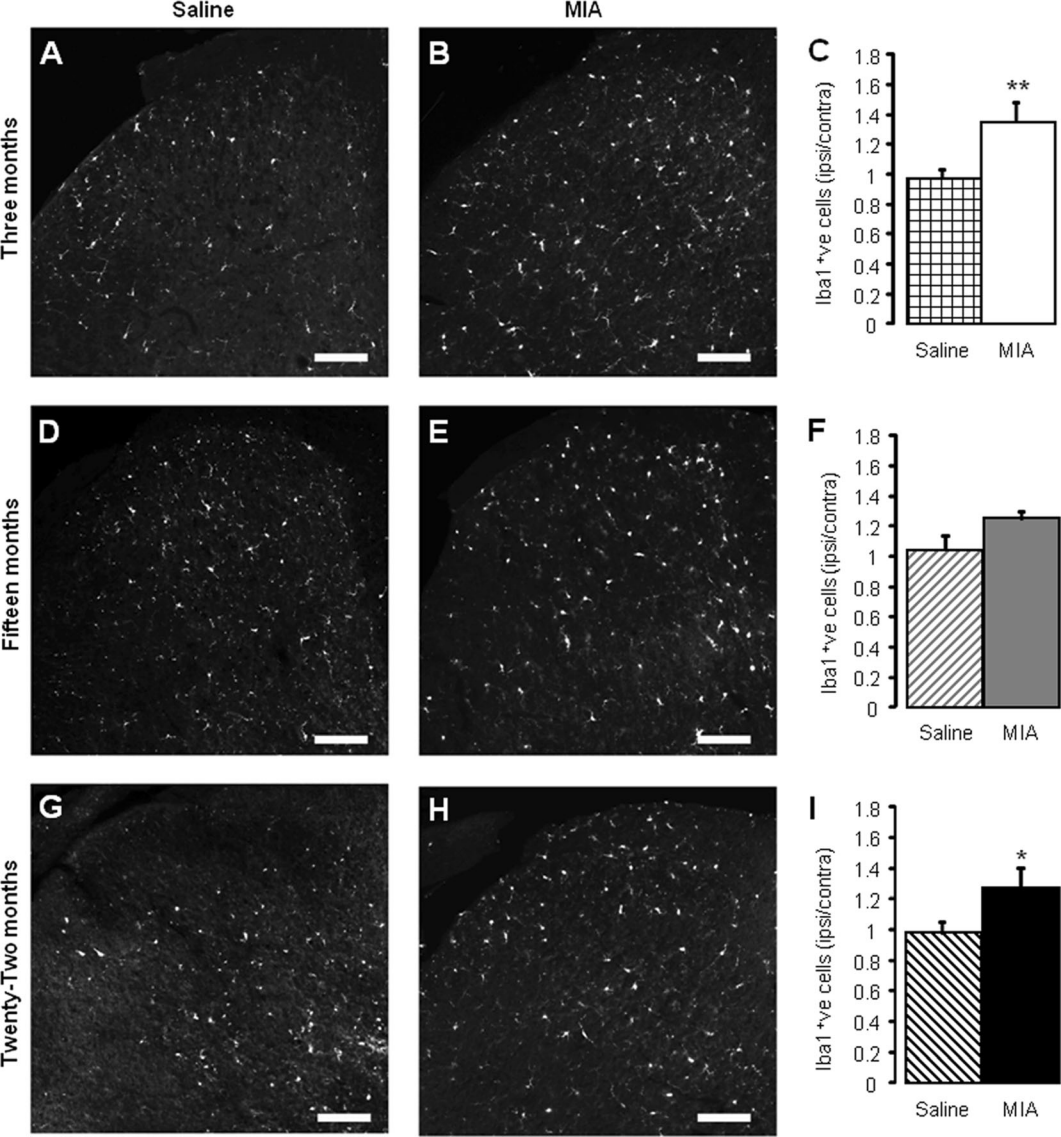
MIA-associated microglial response is attenuated in aged mice. Iba-1 immunoreactivity in L5 ipsilateral dorsal horn in saline (**a**, **d**, **g**) and MIA-treated (**b**, **e**, **h**) mice 28 days after intra-articular administration in 3- (**a**, **b**), 15- (**d**, **e**) and 22- (**g**, **h**) month-old mice. *Scale bars* = 100 μm. Quantification of Iba-1 immunoreactivity in saline and MIA-treated dorsal horn 28 days after administration in 3- (**c**), 15- (**f**) and 22- (**i**) month-old mice. Data shown as mean ± SEM, **p < 0.01, *p < 0.05 compared to age-matched saline group, two-way ANOVA, post hoc Student Newman-Keuls, *n* = 4–5 spinal cords per group

Spinal changes associated with intra-articular injection of MIA include a microglial response in the ipsilateral dorsal horn associated with an increased primary afferent input (Ogbonna et al. 2013). In order to determine whether ageing has an effect on said microglial response, spinal cord tissue from 3-, 15- and 22-month-old mice was immunohistochemically assessed 28 days after MIA/saline administration (Fig. 4). Intra-articular injection of MIA in 3-month-old mice was associated with a significant increase in Iba-1 expressing microglia within the ipsilateral lumbar dorsal horn compared with age-matched saline controls (Fig. 4a–c). The ipsilateral expression of Iba-1 in the dorsal horn of aged 15- (Fig. 4d–f) and 22-month (Fig. 4g–i)-old mice was slightly enhanced after MIA injection in the knee compared to age-matched saline treatment animals; however, this microglial response failed to reach significance in the 15-month-old dorsal horn.

Taken together, these data suggest that the development of the MIA model of OA is altered in aged mice such that (i) weight bearing asymmetry is attenuated; (ii) the early, inflammation-related mechanical hypersensitivity is blunted; (iii) associated spinal microgliosis is reduced. These age-related differences in behaviour and spinal plasticity are observed despite similar effects of MIA within the knee joint itself, thus suggesting that aged mice display an impaired response to the peripheral injection of an inflammatory agent.

### Age-related effects on acute inflammatory pain

**Fig. 5:**
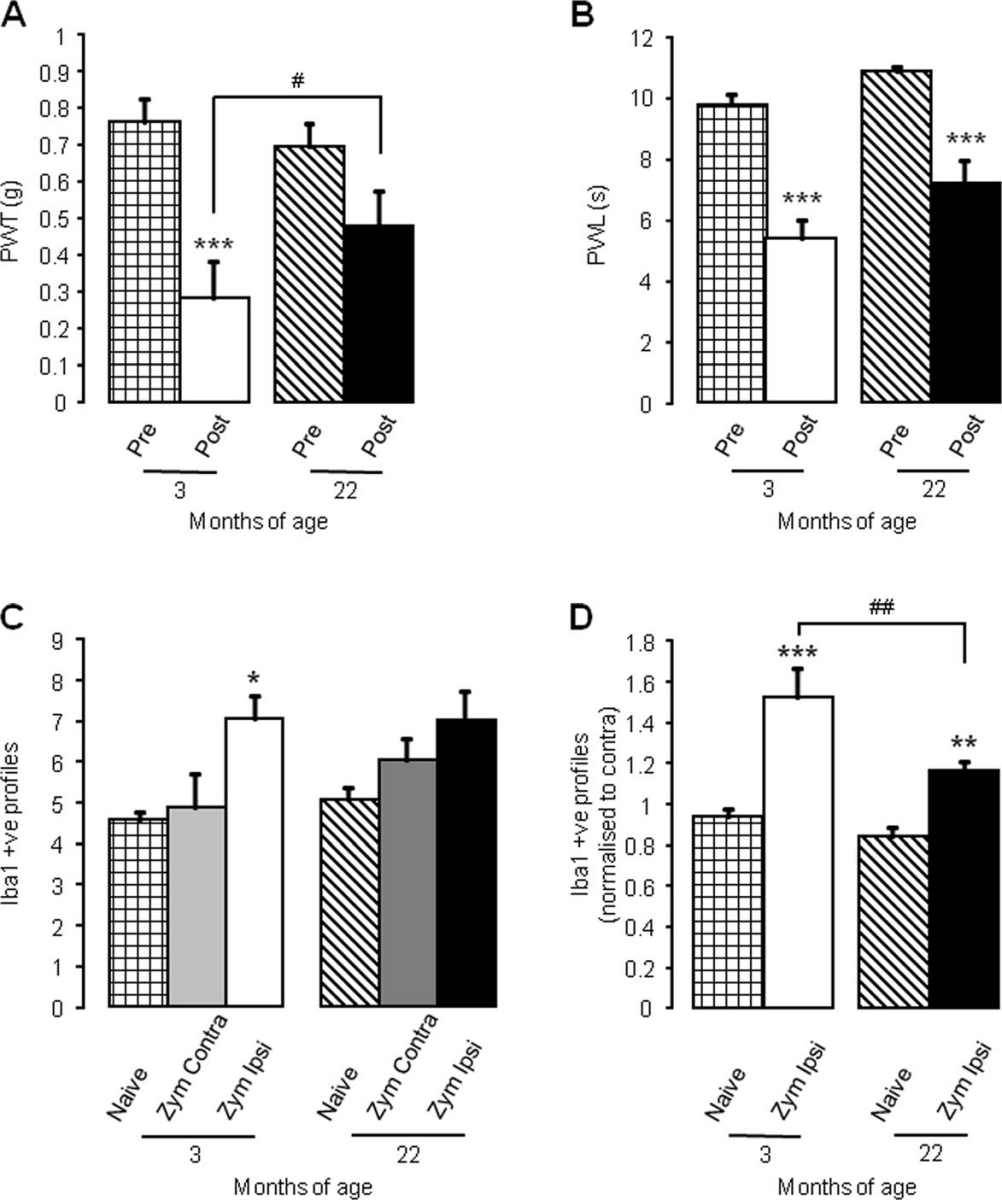
Zymosan-induced nocifensive behaviour and microglial response are attenuated in aged mice (**a**–**b**); mechanical withdrawal responses (**a**) and thermal withdrawal latencies (**b**) of ipsilateral hind paws were measured before (pre) and 24-h post-intra-plantar injection of zymosan (Zym; 20 μL; 0.2 mg/mL). Data shown as mean ± SEM, ***p < 0.001, **p < 0.01, compared to baseline (pre) threshold, two-way ANOVA, post hoc Student Newman-Keuls, *n* = 5 mice per group. **c** Quantification of Iba-1 immunoreactivity (per 10^4^ μm^2^) in ipsilateral and contralateral lumbar (L4 and L5) dorsal horn 24 h after intraplantar zymosan administration (Zym; 20 μL; 0.2 mg/mL) compared to naive dorsal horn from age-matched controls. **d** Quantification of ipsilateral Iba-1 immunoreactivity is normalised to contralateral levels. Data shown as mean ± SEM, **p < 0.01, *p < 0.05, compared to age-matched naive, #*p* < 0.05 compared to indicated group, two-way ANOVA post hoc Student Newman-Keuls, *n* = 5 spinal cords per group

As the initial inflammatory phase of MIA was attenuated in aged mice, we examined the zymosan model of inflammatory pain in 3- and 22-month-old mice to determine if ageing affects the development of acute inflammation. We have previously shown that intra-plantar injection of zymosan produces hypersensitivity to noxious stimuli and dorsal horn microglial activation 24 h after administration (Clark et al. 2007; Staniland et al. 2010). As such, mechanical and thermal thresholds were assessed prior to and 24-h post-zymosan administration followed by immunohistochemical analysis of the lumbar spinal cord. As previously observed (Staniland et al. 2010), administration of zymosan to the hind paw of 3-month-old mice produced both a reduction in PWT to mechanical stimuli indicative of mechanical hypersensitivity (Fig. 5a) and a reduction in paw withdrawal latencies (PWL) to thermal stimuli, indicative of thermal hyperalgesia (Fig. 5b). In 22-month_old mice, however, intra-plantar zymosan did not result in a significant reduction in PWT to mechanical stimuli but did significantly lower PWL to thermal stimuli (Fig. 5a, b). There was no significant difference for both PWL and PWT between ages post-zymosan injection irrespective of differences to their own baseline (Fig. 5a). The nocifensive behaviour produced in 3-month-old mice due to zymosan administration was greater than the overall behavioural results seen in the aged mice. This was evidenced by a 61 ± 15 and 44 ± 8 % reduction in PWT and PWL, respectively, in 3-month-old mice compared to a 34 ± 10 and 34 ± 6 % reduction in PWT and PWL, respectively, in the aged counterparts. In accordance with previous findings (Sweitzer et al. 1999; Clark et al. 2007; Staniland et al. 2010), intra-plantar zymosan induced a significant increase in Iba-1 expressing microglial cells in the dorsal horn of 3-month-old mice (Fig. 5c); 22-month-old mice, however, did not show a significant increase when raw data averages were analysed (Fig. 5d). Microglial cell numbers in the dorsal horn of 22-month-old mice were slightly higher than in 3-month mice (Fig. 5c). When the ipsilateral microglial number was normalised to that of the contralateral dorsal horn, a significant increase in microglial number was observed in 22-month-old mice, but still to a lesser extent to that observed in 3-month-old mice (Fig. 5d).

These data suggest that in the MIA model of OA, aged mice display attenuated weight bearing changes, an attenuation of early inflammation-related mechanical hypersensitivity and reduced spinal microgliosis. Similarly, in a model of acute inflammation, aged mice develop less mechanical hypersensitivity and a microglial response which is less robust than in young adults.

## Discussion

The data obtained in this study demonstrate that ageing is associated with changes in the development of nocifensive behaviours in the MIA model of OA. Specifically, 22-month-old mice displayed reduced weight bearing asymmetry, with both 15- and 22-month old mice developing less severe early phase mechanical hypersensitivity compared to young adult mice. An age-related reduction in MIA-associated microgliosis in the dorsal horn of the spinal cord was also observed. These alterations are apparent despite no gross differences when comparing knee pathology between age groups. Furthermore, in a model of acute inflammation, 22-month-old mice developed less mechanical hypersensitivity than 3-month-old mice, which was coupled with a reduction in microglial activation. The lack of obvious correlation between knee pathology after MIA administration and altered behavioural responses with age mirrors the subpopulation of OA patients with reported knee pain but minimal knee pathology, and vice versa. Notwithstanding the limitation of the current study in which the extent of joint damage was not quantified, our data provide support for significant contribution of central plasticity mechanisms to OA pain and furthermore, that age-related changes within the CNS could affect the manifestation of different components of OA pain (Ogbonna et al. 2013; Rahman and Dickenson 2015).

When assessing measures of ongoing pain using weight bearing analysis and evoked referred allodynia using von Frey filaments applied to the hind paw, effects of age on the MIA model of OA are evident. Whilst 15-month-old mice displayed weight bearing asymmetry immediately from 3 days after MIA injection and to a greater degree than 3-month-old counterparts, this pain behaviour was significantly reduced in 22-month-old mice and completely absent for the early phase of the study. In a study by Gagliese and Melzack, 3- and 24-month-old animals received intra-plantar injections of the inflammogen formalin and had similar pain scores, which were significantly less than for 18-month-old animals who also received formalin injections (Gagliese and Melzack 1999). They suggest that sensitivity to persistent pain may peak at midlife, which would support the data presented here. A possible explanation could be neurochemical and neuroanatomical changes that occur at mid-life and possibly alter the response to tonic painful stimulation. These include the onset of disinhibition by the degeneration of endogenous descending inhibitory pathways (Iwata et al. 1995) and a life-time peak in the dorsal root ganglia neuronal number and size (Devor 1991), which could lead to increased sensitivity to ongoing pain at mid-life. With advancing age, there may be further changes within the nociceptive pathway that impair nociceptive transmission. In the life span of a mouse, it has been reported that from 12 to 20 months of age, the mouse peripheral nerve shows only mild signs of age-related changes which progressively appear, with number and density of nerve fibres beginning to decrease (Ceballos et al. 1999). This type of age-related change would be expected to result in reduced nociceptive transmission to the spinal cord. However, it may be that the peripheral nerve changes are not robust enough to counteract the possible increased excitability within the dorsal horn, as a result of age-related disinhibiton (Iwata et al. 1995). From 20 months of age, however, nerves show a general disorganisation, marked fibre loss and decreased density of nerve fibres (Ceballos et al. 1999), which may be sufficient to result in impaired nociceptive transmission.

The pattern of age-related effect on ongoing pain determination was not replicated in the assessment of referred mechanical allodynia. Both 15- and 22-month old mice exhibited attenuated early phase responses to MIA injection, with mechanical hypersensitivity developing later compared to 3-month-old mice. This early phase pain response has been reported to be sensitive to NSAIDs (Bove et al. 2003; Pomonis et al. 2005; Vonsy et al. 2009), and it is generally accepted that it occurs as a result of MIA-induced inflammation at the joint. The absence of this response in aged mice suggests an age-related reduction in the peripheral inflammatory response. Interestingly, aged mice still developed significant mechanical hypersensitivity in the late phase of the MIA model, suggesting that there is still the capacity for a large enough peripheral drive to activate dorsal horn neurons; this increased peripheral drive may be coming from sources other than nociceptive fibres. Wu and Henry, reported changes in electrophysiological properties of non-knee joint afferents and Aβ fibre non-nociceptive primary afferent neurons after surgical induction of OA which may be involved in the pathogenesis of OA pain (Wu and Henry 2010). This also provides support for non-articular factors contributing to OA pain and may offer some explanation for the lack of correlation between joint pathology and severity of pain, as well as incidence of pain in areas spatially distinct from the OA affected joint. The mechanisms mediating Aβ fibre responses remain unclear.

Peripheral inflammatory stimuli trigger central microglial responses (Svensson et al. 2003; Clark et al. 2007; Staniland et al. 2010) with consequent proliferation and release of pro-inflammatory mediators. MIA-induced microgliosis was less robust with age, supporting the concept that central inflammatory responses, as well as peripheral inflammatory responses, are diminished with age. However, this hypothesis is inconsistent with the fact that a significant behavioural response is still seen at the same time point in aged mice.

In summary, here, we provide evidence for an age-related impairment of MIA-induced pain responses in terms of nocifensive behaviour. Furthermore, we provide initial evidence that microglial response to peripheral damaging stimuli may be dysfunctional with advancing age.
